# Overexpression of microRNA-145 enhanced docetaxel sensitivity in breast cancer cells via inactivation of protein kinase B gamma-mediated phosphoinositide 3-kinase -protein kinase B pathway

**DOI:** 10.1080/21655979.2022.2068756

**Published:** 2022-05-01

**Authors:** Ying Zhou, Wei Cai, Hailin Lu

**Affiliations:** Department of Oncology, Suzhou Ninth People’s Hospital, Suzhou, Jiangsu, China

**Keywords:** Breast cancer, docetaxel, sensitivity, miR-145, AKT3

## Abstract

Chemoresistance is a major challenge for the treatment of breast cancer (BC). Previous studies showed that miR-145 level decreases in chemoresistant BC tissues. Nevertheless, the biological function of miR-145 on docetaxel resistance of BC cells remains unclear, which is what our research attempted to clarify. RT-qPCR analyzed miR-145 level, and cell viability and colony formation assays assessed the impact of miR-145 on docetaxel resistance. Molecular mechanisms of miR-145-mediated docetaxel sensitivity were examined by Luciferase reporter assay and Western Blot assessed the function of AKT3 and PI3K/AKT signaling. Our research found that miR-145 expression presented significant downregulation in docetaxel-resistant BC cells. Meanwhile, miR-145 overexpression facilitated the docetaxel sensitivity of BC cells *in vivo* and *in vitro*, while the miR-145 inhibitor decreased the sensitivity of BC cells to docetaxel. We also observed that miR-145 inhibited docetaxel resistance mainly via downregulation of the AKT3 expression and further inhibited PI3K/AKT pathway. To conclude, this research provides a novel strategy for improving chemosensitivity through the newly identified miR-145-AKT3/PI3K-AKT signaling pathway in BC.

## Graphical Abstract

**Figure:**
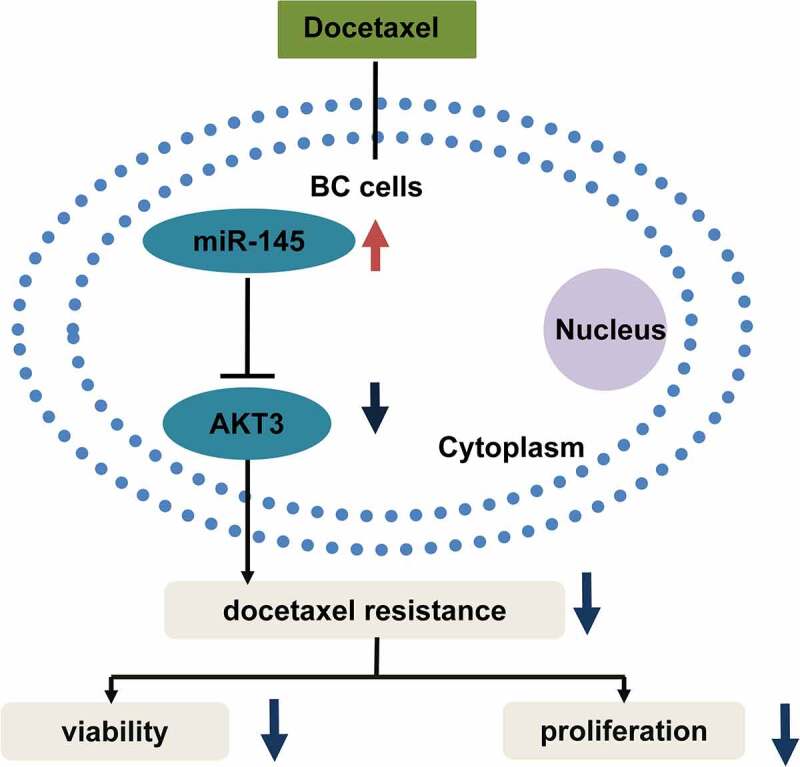

## Highlights

•The level of miR-145 was decreased in docetaxel resistant BC cells.

•MiR-145 alleviated the docetaxel resistance of BC cells via PI3K/AKT pathway.

•MiR-145 targeted AKT3 directly and inhibited PI3K/AKT pathway.

•AKT3 and PI3K/AKT inhibition enhanced the sensitivity of BC cells to docetaxel.

## Introduction

Breast cancer (BC) ranks the first in prevalent tumors among females and is the second leading cause of high mortality in women in the United States [1]. The treatments for BC include surgical treatment, radiation therapy, chemotherapy, and endocrine therapy [2,3]. Attempts have also been made to curb cancers with fungal-derived materials and drug delivery coupled with nanobiomaterials in the recent decades [4,5]. Among them, the most commonly used one is chemotherapy [6]. Chemotherapy utilizes the different drug sensitivities of cells to accurately inhibit the cell proliferation. Cancer cells are more sensitive to chemotherapy for their exuberant proliferation than slowly proliferating cells and are chemosensitive tissue within the body. However, chemoresistance is the biggest obstacle to such treatment [7,8]. Docetaxel, a kind of taxanes, has potent antitumor activity in progression and is one of the most active drugs used in BC treatment [9,10]. Hence, the docetaxel chemoresistance of BC cells is worth investigating.

MicroRNAs (miRNAs), a subgroup of endogenously processed non-coding RNAs, inhibit their target gene level and translation [11]. The exceptional stability of miRNAs has presented them as biomarkers with high specificity and sensitivity, which is crucial for early diagnosis [12–14]. Researchers have examined the effects of miRNAs on chemoresistance in multiple cancers [15,16]. For instance, miR-199a inhibits glioma tumor growth and chemoresistance via negatively regulating kinase suppressor of ras 1 [17]. MiR-133b overexpression decreases tumor cell stemness and overrides chemoresistance to 5-fluorouracil and oxaliplatin in colorectal cancer [18]. In BC, the knockdown of miR-141 abrogates docetaxel resistance of BC cells through inhibiting EIF4E [19].

As a newly discovered microRNA, miR-145 locates on chromosome 5q32 with 4.08 KB, and presents significant downregulation in a variety of tumors. Previously, miR-145 was found to be significantly down-regulated in gastric cancer SGC-7901 cells. Besides, miR-145 expression was prominently lower in lung cancer patients, also serving as a potential diagnostic biomarker. Moreover, in prostate cancer, bladder cancer and hepatocellular carcinoma, miR-145 upregulation inhibits tumor cell proliferation and promotes apoptosis [17–19]. Recently, miR-145 has been reported to present downregulation in BC patients [20]. More importantly, miR-145 prominently decreased in chemoresistant BC tissues [21]. However, the biological function of miR-145 to docetaxel resistance remains unclear.

PI3K/AKT signaling pathway regulates different biological behaviors, including cell growth, cell apoptosis, protein synthesis, and glycolytic metabolism [22–25]. PI3K/AKT is a major pathway, and its regulatory activation has been proved to be the reason of aberrant growth of tumor cells and their drug resistance [26,27]. In BC, Luo et al. reported that PI3K/AKT signaling activation is implicated in regulating multidrug resistance [28]. Meanwhile, Huang et al. elucidated that PI3K/AKT pathway contributes to docetaxel resistance in breast cancer [29]. However, whether miR-145 could interfere PI3K/AKT pathway to regulate docetaxel sensitivity in BC still remains obscure.

Herein, we test the hypothesis that the elevation of miR-145 expression promotes docetaxel sensitivity of BC cells by inactivating the PI3K/Akt pathway. The aim of this study is to investigate innovatively the effect of miR-145 on docetaxel resistance of BC cells both *in vitro* and *in vivo*. Moreover, the target role of AKT3 toward miR-145 and its influence on the docetaxel resistance of BC cells were verified. Thus, our research showed the potential of miR-145/AKT3/PI3K-Akt axis in modulating the docetaxel resistance of BC cells.

## Materials and methods

### Cell culture and transfection

BC cell lines (MCF7 and MDA-MB-231) were originally purchased from ATCC (Manassas, USA). Docetaxel resistant cell lines, MCF7/docetaxel (DTX), and MDA-MB-231/DTX cell lines were developed following sequential exposure to docetaxel (Selleck Chemical, Houston, USA) at increasing concentrations as previously described [30]. The cells were maintained in RPMI-1640 supplemented with 10% fetal bovine serum at 37°C in a humidified atmosphere containing 5% carbon dioxide. miR-145 mimics, inhibitors, or negative control (NC) mimic and inhibitor NC (Ambion, Austin, TX, USA) were transfected into BC cells using Lipofectamine RNAiMAX Reagent (Invitrogen, Carlsbad, CA, USA) following the manufacturer’s protocol. All sequences are available in Table 1.

**Table 1.** Oligonucleotide sequences

Oligonucleotides	Sequences
miR-145 mimic	5’-GUCCAGUUUUCCCAGGAAUCCCU-3’
miR-145 mimic control	5’-CCAAGCCAUUUCGUCGCCUGUAU-3’
miR-145 inhibitor	5’-AGGGAUUCCUGGGAAAACUGGAC-3’
miR-145 inhibitor control	5’-CGUCCGUAAGAAGAUCAGUGGGA-3’

### RNA isolation and real-time quantitative reverse transcription-PCR

Total RNA was isolated from cultured cells using QIAzol Lysis Reagent (79,306). To analyze the expression of miR-145, reverse transcription and qRT-PCR were performed using miScripIIRT kit (QIAGEN, Hilden, Germany) and miScript SYBR® Green PCR kit according to the manufacturer’s protocol. U6 or β-actin was used as the internal control. The sequences of primers are listed in Table 2.

**Table 2.** Primers for qRT-PCR

Primer name	Primer sequence
miR-145 Forward	5’-CTCAACTGGTGTCGTGGAGTCGGCAATTCAGTTGAGAGGGATTC-3’
miR-145 Reverse	5’-ACACTCCAGCTGGGGTCCAGTTTTCCCAGGA-3’
U6 Forward	5’-CTCGCTTCGGCAGCACA-3’
U6 Reverse	5’-AACGCTTCACGAATTTGCGT-3’
AKT3 Forward	5’-TGTGGATTTACCTTATCCCCTCA-3’
AKT3 Reverse	5’-GTTTGGCTTTGGTCGTTCTGT-3’
β-actin Forward	5’-GACTTAGTTGCGTTACACCCTTTCTTG-3’
β-actin Reverse	5’-GACTGCTGTCACCTTCACCGTTC-3’

### Cell viability assay and colony formation assay

Cell viability assay was performed using BC cells treated with or without docetaxel for 48 h at different concentrations, as previously described [31]. In addition, we conducted the colony formation assay using BC cells treated with or without docetaxel (5 nM) as previously described [31].

### Luciferase reporter assay

The pMIR-REPORT luciferase vectors containing a wide type or mutant 3’-UTR sequences of AKT3 were obtained from GenePharma (Shanghai, China). The mutant 3’-UTR sequences of AKT3 carry mutated putative binding sites of miR-145. MCF7 cells were co-transfected with a luciferase reporter plasmid, a-galactosidase vector, and a miR-145 mimic to perform luciferase reporter assays, as previously described [32].

### Western blot

Total protein was extracted from cells using radioimmunoprecipitation assay buffer (Beyotime, China) and the protein was separated on 10% sodium dodecyl sulfate-polyacrylamide gel electrophoresis, as previously described [33]. The antibodies used were as follows: anti-AKT3 (Abcam, Cambridge, MA, USA; ab2157); anti-p-AKT (Abcam; ab8805); anti-P-AKT (Abcam; ab8805; ab192623); and anti-GAPDH (Abcam; ab9485). Original Western Blot images were provided in Supplementary file 1.

### Chemosensitivity assay in vivo

miR-145-overexpressed lentivirus (LV-miR-145) was obtained from GenePharma (Shanghai, China). MCF7/DTX cells were transduced with LV-miR-145 at MOI of 10 as previously described [34]. Eighteen to twenty-two g BALB/c nude mice were purchased from Vital River Laboratory Animal Technology Co., Ltd. (Beijing, China). All animal experiments were approved by the Institutional Animal Care and Use Committee of Suzhou Ninth People’s Hospital (No.2020-LL-0015A). LV-miR-145-infected MCF7/DTX cells (5 × 10^6^) were injected subcutaneously into the right-dorsal flanks. All mice were treated with DTX (10 mg/kg) twice per week for 2 weeks. The length and width of the model tumor were measured with calipers at different time points. The tumor volume was calculated as the following formula: volume = [(length × width^2^)/2]. At the end of this experiment, the mice were euthanized. Tumor weights were determined after isolation. A copy of the ethical approval certificate was provided in Supplementary file 2.

### Statistical analysis

Each experiment was repeated 3 times. Data were presented as mean ± standard deviation (SD). The difference between the two groups was analyzed with Student’s t-test. Comparisons among multiple groups were measured using a one-way analysis of variance with Dunnett’s and Tukey’s post hoc tests. For multiple comparison with two variables, a two-way analysis of variance was conducted. P < 0.05, P < 0.01, or P < 0.001 were considered statistically significant.

## Results

In this work, the role of miR-145 in mediating docetaxel sensitivity was explored, based on normal and docetaxel-resistant BC cell lines and samples from nude mice. It was found that upregulation of miR-145 enhanced docetaxel sensitivity of BC cells via inhibition of PI3K/AKT pathway. Overexpression of miR-145 alleviated the docetaxel resistance of BC cells. MiR-145 targeted AKT3 directly and inhibited PI3K/AKT pathway. Thus, our study provided new insights into miR-145 as a potential molecular target for breaking chemoresistance of BC.

### The level of miR-145 was decreased in docetaxel resistant BC cells

**Figure 1.**
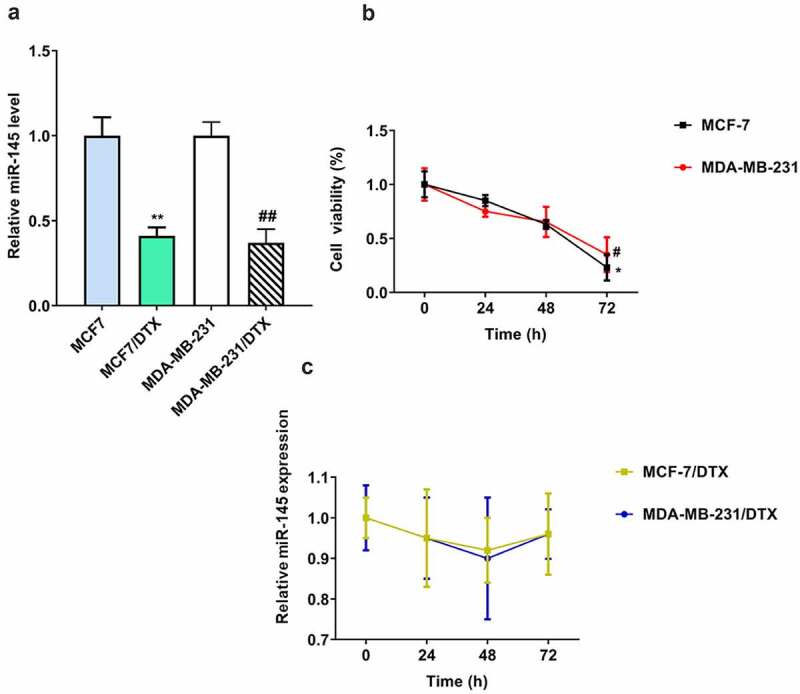
Expression of miR-145 docetaxel-resistant BC cell lines. (a) MIR-145 expression was analyzed in MCF-7 and MDA-MB-231 cells and docetaxel-resistant (MCF-7/DTX and MDA-MB-231/DTX) cells via qRT-PCR. (b, c) MIR-145 expression was analyzed in MCF-7 and MDA-MB-231 cells (b) and docetaxel-resistant (MCF-7/DTX and MDA-MB-231/DTX) (c) cells after treated with docetaxel (5 nM) via qRT-PCR. Values were expressed as means (SD). *P < 0.05, **P < 0.01, #P < 0.05, ##P < 0.01.

As shown in (Figure 1a), the miR-145 expression was significantly downregulated in MCF7/DTX and MDA-MB-231/DTX cells compared with MCF-7 and MDA-MB-231 cells. In addition, to explore the effect of docetaxel on miR-145 expression in BC cells, both BC and BC/DTX cells were treated with docetaxel for 72 h. The miR-145 level was gradually decreased over time in MCF-7 and MDA-MB-231 cells (Figure 1b). In contrast, docetaxel treatment had no influence on the expression of miR-145 in MCF7/DTX and MDA-MB-231/DTX cells (Figure 1c).

### miR-145 overexpression enhanced the sensitivity of BC/DTX cells to docetaxel

**Figure 2.**
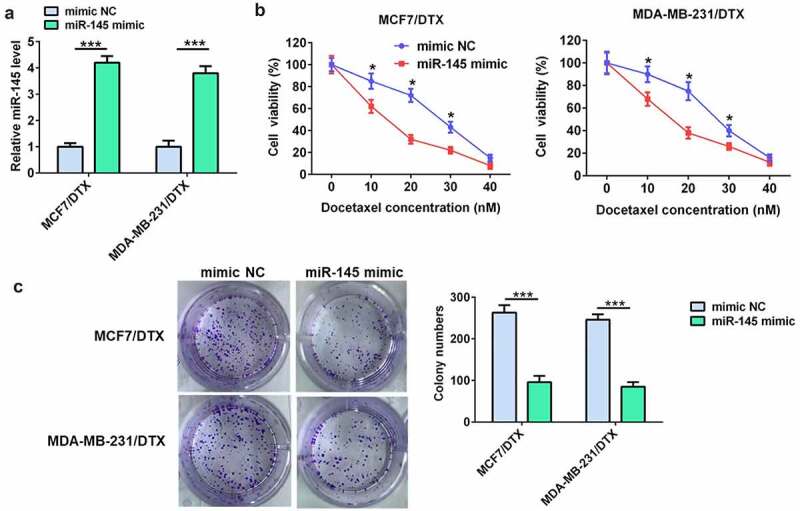
miR-145 overexpression inhibited BC cell resistance to docetaxel. (a) qRT-PCR analysis of miR-145 expression in MCF-7/DTX and MDA-MB-231/DTX cells after miR-145 mimic transfection. (b) Effect of miR-145 overexpression on the cell viability of MCF-7/DTX and MDA-MB-231/DTX cells cultured in the presence of docetaxel. (c) A clonogenic survival assay was performed using miR-145-overexpressed MCF-7/DTX and MDA-MB-231/DTX cells cultured in the presence of docetaxel. Values were expressed as means (SD). *P < 0.05, ***P < 0.001.

To demonstrate the role of miR-145 in docetaxel resistance of BC cells, MCF7/DTX and MDA-MB-231/DTX cells were transfected with miR-145 mimic. The RT-qPCR results showed that miR-145 mimic significantly up-regulated miR-145 level in both MCF7/DTX and MDA-MB-231/DTX cells (Figure 2a). The cell viability and colony formation assay indicated that miR-145 mimic considerably enhanced the docetaxel sensitivity of MCF7/DTX and MDA-MB-231/DTX cells (Figure 2b,c).

### miR-145 inhibitor decreased the sensitivity of BC cells to DTX

**Figure 3.**
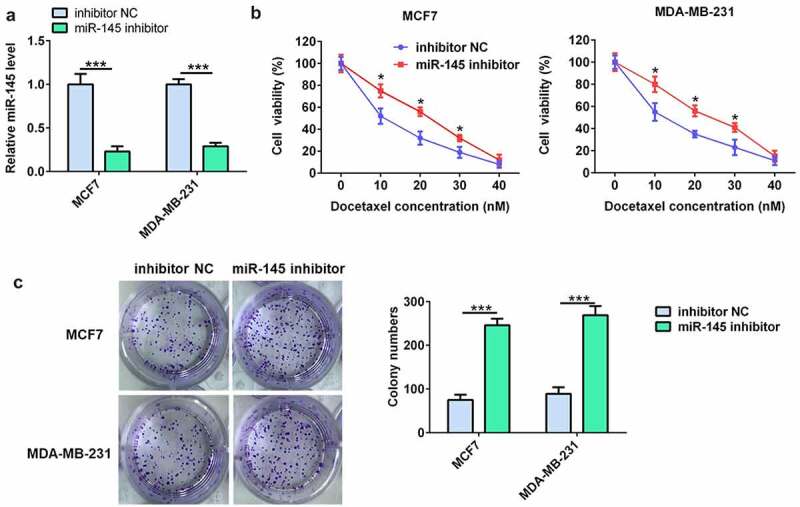
miR-145 inhibitor increased BC cell resistance to docetaxel. (a) qRT-PCR analysis of miR-145 expression in MCF-7 and MDA-MB-231 cells after miR-145 inhibitor transfection. (b) Effect of miR-145 inhibitor on the cell viability of MCF-7 and MDA-MB-231 cells cultured in the presence of docetaxel. (c) A clonogenic survival assay was performed using miR-145-inhibitor MCF-7 and MDA-MB-231 cells cultured in the presence of docetaxel. Values were expressed as means (SD). *P < 0.05, ***P < 0.001.

The miR-145 inhibitor was further used to evaluate the effect of miR-145 on docetaxel resistance in BC cells. As shown in (Figure 3a), the RT-qPCR results showed that miR-145 inhibitor significantly downregulated the miR-145 level in MCF7 and MDA-MB-231 cells. Moreover, the results of cell viability and colony formation assay indicated that miR-145 inhibitor prominently decreased the docetaxel sensitivity of MCF7 and MDA-MB-231 cells (Figure 3b,c).

### miR-145 targeted AKT3 and inhibited PI3K/AKT signaling

**Figure 4.**
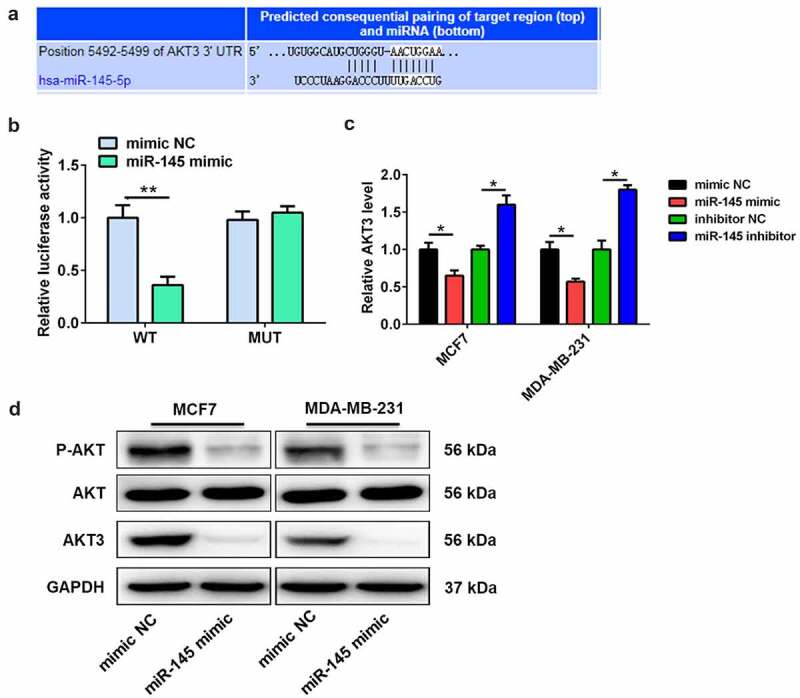
AKT3 is a target of miR-145. (a) Bioinformatics analysis showed the complementary sites of miR-145 in AKT3 3’-UTR. (b) Luciferase activity assay was performed in MCF7 cells co-transfected with either AKT3 wild-type plasmid or AKT3 mutant plasmid and miR-145 mimic. (c) The mRNA expression of AKT3 was detected in MCF-7 and MDA-MB-231 cells after miR-145 mimic or inhibitor transfection. (d) The protein expression of AKT3 and total AKT and the P-AKT level was analyzed in MCF-7 and MDA-MB-231 cells after miR-145 mimic transfection. Values were expressed as means (SD). *P < 0.05, **P < 0.01.

Targetscan 7.2 software was used to investigate the target genes in order to explore the molecular mechanisms of miR-145-mediated docetaxel sensitivity. The interaction targets between miR-145 and AKT3 3ʹUTR are shown in Figure 4a. The luciferase reporter assay showed that miR-145 mimic significantly downregulated the luciferase activity of luciferase reporter plasmid containing wide-type 3ʹUTR of AKT3 compared with mimic NC (Figure 4b), with no effect on the luciferase activity of luciferase reporter plasmid containing with mutant 3ʹUTR of AKT3 (Figure 4b). In addition, the mRNA expression of AKT3 in BC cells was decreased by miR-145 mimic, while miR-145 inhibitor increased the mRNA expression level (Figure 4c). Thus, miR-145 mimic reduced the protein level of AKT3 in BC cells (Figure 4d). More importantly, miR-145 mimic also decreased the phosphorylation level of AKT (Figure 4d).

### AKT3 knockdown or PI3K/AKT signaling inhibition enhanced the sensitivity of BC/DTX cells to docetaxel

**Figure 5.**
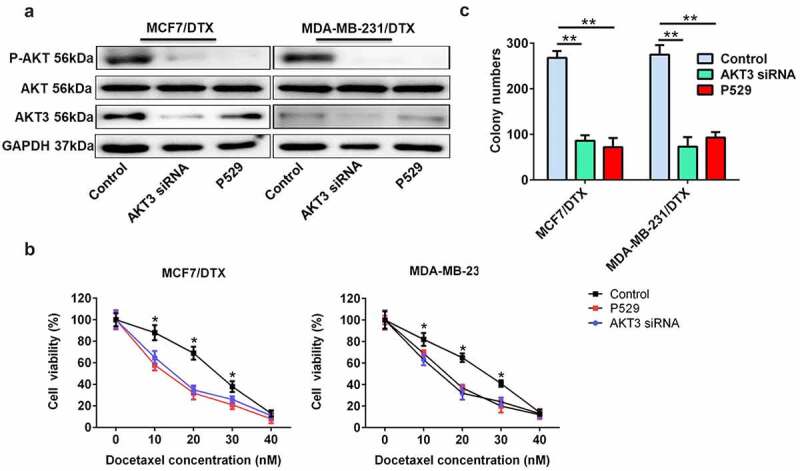
AKT3 knockdown or PI3K/AKT signaling inhibition enhanced the sensitivity of BC/DTX cells to docetaxel. (a) The protein expression of AKT3 and total AKT and the P-AKT level was analyzed in MCF-7/DTX and MDA-MB-231/DTX cells treated with AKT3 siRNA or P529. (b) AKT3 siRNA or P529 treatment reduced the cell viability of MCF-7/DTX and MDA-MB-231/DTX cells cultured in the presence of docetaxel. (c) A clonogenic survival assay was performed in MCF-7/DTX and MDA-MB-231/DTX cells co-treated with AKT3 siRNA or P529 and docetaxel. Values were expressed as means (SD). *P < 0.05, **P < 0.01.

To investigate the effect of AKT3 and PI3K/AKT signaling on docetaxel sensitivity in BC cells, we downregulated the expression of AKT3 with a specific siRNA for AKT3 and inhibited the PI3K/AKT signaling using the P-AKT inhibitor, Palomid 529 (P529) (Figure 5a). The cell viability and colony formation assay showed that both AKT3 siRNA and P529 remarkably enhanced the docetaxel sensitivity of MCF7/DTX and MDA-MB-231/DTX cells (Figure 5b,c).

### miR-145 overexpression enhanced the docetaxel sensitivity in vivo

**Figure 6.**
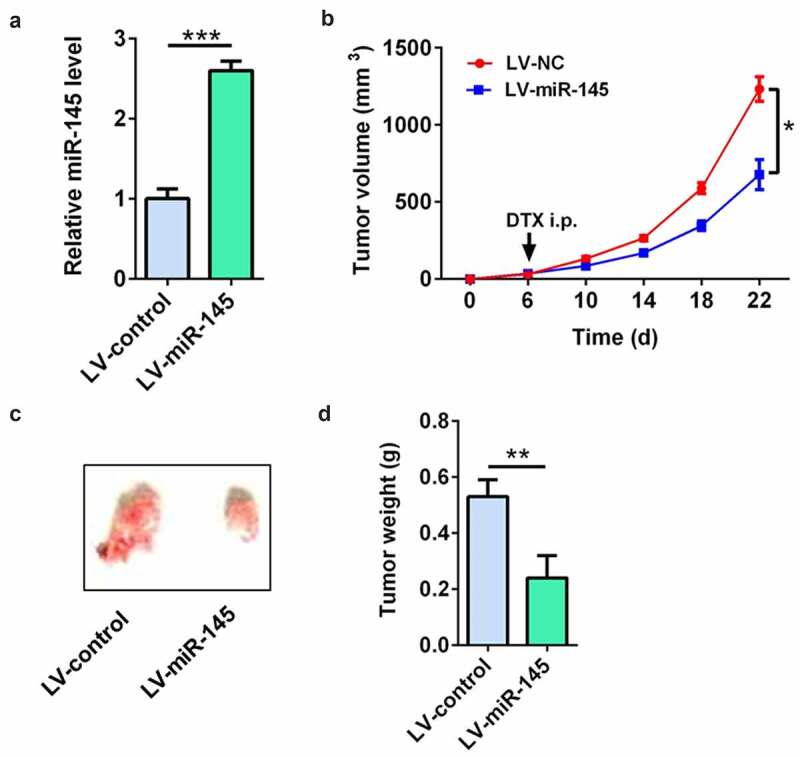
miR-145 overexpression enhanced the docetaxel sensitivity *in vivo*. (a) qRT-PCR analysis of miR-145 expression in MCF-7/DTX cells after LV-miR-145 transduction. (b-d) The tumor volume (b), tumor picture (c) and tumor weight (d) showed that miR-145 overexpression enhanced the docetaxel sensitivity in xenografts. Values were expressed as means (SD). *P < 0.05, **P < 0.001; ***P < 0.001.

LV-miR-145-infected MCF7/DTX cells were used to demonstrate the effect of miR-145 on docetaxel sensitivity of BC cells *in vivo*. The RT-qPCR results showed that the miR-145 level was significantly up-regulated in LV-miR-145-infected MCF7/DTX cells (Figure 6a). The tumor volume, tumor picture, and tumor weight showed that compared with tumors in the LV-NC-infected MCF7/DTX cell group, the tumors were significantly decreased in the LV-miR-145-infected MCF7/DTX cell group after docetaxel treatment (Figure 6(b-d)). These results suggest that miR-145 overexpression enhanced the docetaxel sensitivity *in vivo*.

## Discussion

Docetaxel is a common chemotherapeutic agent for the therapy of numerous tumors, such as BC [35,36]. However, docetaxel resistance results in treatment failure for patients with advanced BC. Therefore, it is necessary to find out the mechanism of docetaxel resistance in BC cells [37]. Herein, miR-145 showed significant downregulation in MCF7/DTX and MDA-MB-231/DTX cells relative to MCF-7 and MDA-MB-231 cells. MiR-145 elevation enhanced BC/DTX cell sensitivity to docetaxel. Notably, we found that miR-145 inhibited docetaxel resistance mainly via downregulating AKT3/PI3K-AKT pathways.

MiR-145, an anti-tumor microRNA [38,39], was validated to participate in multiple BC biological behaviors [40,41]. Jiang et al. found that miR-145 was a negative correction with HBXIP in BC tissues and it suppressed BC cell proliferation via targeting HBXIP [42]. Sequencing data depict that miR-145 elevation triggers whole-transcriptome alteration and further results in the decreased BC cell proliferation/migration/invasion [43]. Moreover, miR-145 was reported to participate in regulating chemoresistance in BC. Gao et al. reported that miR-145 bound to the multidrug resistance-associated protein 1 (MRP1) 3ʹUTR and further downregulated MRP1 expression in BC cells. MiR-145 overexpression elevated BC cell sensitivity to doxorubicin and doxorubicin chemotherapy [44]. In our study, miR-145 level decreased in docetaxel-resistant BC cells. Docetaxel treatment gradually decreased miR-145 level over time in BC cells, while it had no influence on miR-145 level in docetaxel-resistant BC cells. This drug-induced change in miRNA expression was novelly found and could bring new considerations to pharmacy. MiR-145 upregulation enhanced BC/DTX cell sensitivity to docetaxel. In the same way, miR-145 inhibitor decreased BC cell sensitivity to docetaxel. More importantly, miR-145 overexpression enhanced the docetaxel sensitivity *in vivo*, which accelerated the pace of clinical use. Our findings suggested that miR-145 overexpression enhances the sensitivity of BC cells to docetaxel.

We attempted to clarify the mechanism of such miR-145-mediated modulation on docetaxel resistance in BC cells. AKT3 was predicted to be targeted by miR-145. AKT3 is one of the three mammalian Akt isoforms (Akt1, Akt2, and Akt3) [45]. In triple-negative breast cancer, knockdown of AKT3 inhibits tumor spheroid growth in three-dimensional (3D) and in xenografts via significant elevation of cell-cycle inhibitor p27 [46]. AKT3 depletion increases the expression of S100A4 and promotes both migrations *in vitro* and metastasis formation *in vivo* [47]. Moreover, in AKT inhibitor-resistant cells, AKT3 was found to be markedly up-regulated, promoting BC cell resistance to AKT inhibitors, such as MK2206 [48]. However, the underlying molecular mechanism for AKT3-mediated docetaxel resistance in BC cells has not yet been defined. In this study, we found that AKT3 knockdown or PI3K/AKT signaling inhibition facilitated BC/DTX cell sensitivity to docetaxel, suggesting that AKT3 is important for docetaxel resistance in BC cells. Such an effect was further analyzed, and it is shown that AKT3 was directly targeted by miR-145. Moreover, miR-145 mimic reduced mRNA expression of AKT3 in BC cells, while miR-145 inhibitor increased the mRNA expression level. Additionally, miR-145 mimic reduced the phosphorylation level of AKT. These findings depict that the miR-145-mediated improvement of docetaxel sensitivity in BC cells is accomplished via AKT3/PI3K-AKT signaling inhibition.

In summary, our findings could provide a novel strategy for improving chemosensitivity in BC cells through the newly identified miR-145–AKT3/PI3K–AKT axis, which will provide a viable therapy for BC in clinical practice.

## Conclusion

In conclusion, here we determined the role of miR-145 in docetaxel resistance of BC cells both *in vivo* and *in vitro*. Specifically, we verified our speculation that miR-145 modulates docetaxel sensitivity of BC cells by inactivating PI3K/AKT pathway and AKT3 is directly targeted by miR-145. However, whether it functions the same in *in vivo* assay with BC patients still needs further clinical study. There are also two directions of use to improve the present challenges in BC: to detect the expression level of miR-145 for BC prognosis and to overexpress miR-145 to increase docetaxel sensitivity of BC cells.

## Supplementary Material

Supplemental Material
