# Supplementary material for: Overexpression of microRNA-145 enhanced docetaxel sensitivity in breast cancer cells via inactivation of protein kinase B gamma-mediated phosphoinositide 3-kinase -protein kinase B pathway
Source: Bioengineered. 2022 May 1;13(4):11310–20. doi: 10.1080/21655979.2022.2068756 (PMC9278436; doi:10.1080/21655979.2022.2068756)
Supplement: Supplemental Material [file KBIE_A_2068756_SM7437.zip › supplementary/supp pdf.pdf]

Medical Ethics Review Report

Suzhou Ninth People's Hospital applies to conduct a project "miR-145 enhanced the docetaxel sensitiveness in breast cancer cells by suppressing AKT3-mediated PI3K-Akt pathway" for scientific research. The Institutional Review Board of the Suzhou Ninth People's Hospital censored the probably associated medical ethics issue in the project.

Name of Project:

miR-145 enhanced the docetaxel sensitiveness in breast cancer cells by suppressing AKT3-mediated PI3K-Akt pathway

Institution in Charge:

Suzhou Ninth People's Hospital (No. 2020-LL-0015A)

Leader of Project:

Hailin Lu

Comments of review:

The contents of this study are under full compliance with government policy and the Declaration of Helsinki. There are no conflicts between results and the contents of project.

Conclusion:

The Institutional Review Board of the Suzhou Ninth People's Hospital approve this project to be carried according to designed.

February 26, 2020
